# Adaptive Multiple-Attribute Scenario LoRA Merge for Robust Perception in Autonomous Driving

**DOI:** 10.3390/s26041336

**Published:** 2026-02-19

**Authors:** Ryosuke Kawata, Joonho Lee, Yanlei Gu, Shunsuke Kamijo

**Affiliations:** 1 Graduate School of Interdisciplinary Information Studies, The University of Tokyo, Tokyo 113-0033, Japan; leejoonho@kmj.iis.u-tokyo.ac.jp; 2 Graduate School of Advanced Science and Engineering, Hiroshima University, Hiroshima 739-8527, Japan; guyanlei@hiroshima-u.ac.jp; 3 Interfaculty Initiative in Information Studies, The University of Tokyo, Tokyo 113-0033, Japan; kamijo@iis.u-tokyo.ac.jp

**Keywords:** autonomous driving, parameter-efficient fine-tuning, model merging

## Abstract

Perception models for autonomous driving are predominantly trained on clear, daytime data, leaving their performance under rare conditions—particularly in multiple-attribute (joint weather–lighting) conditions such as night × rainy or night × snowy—an open challenge. To address this, we propose a parameter-efficient fine-tuning (PEFT) framework that dynamically applies lightweight, scenario-specific Low-Rank Adaptation (LoRA) experts. At its core, our method features an adaptive pipeline that dynamically determines the LoRA experts to apply based on the encountered environmental conditions. We validate our framework on a unified semantic segmentation benchmark (MUSES, BDD100K, and Cityscapes) covering six scenarios (day/night × weather). Our approach improves the mIoU by up to 3.23 points over a strong baseline in single-attribute settings, and in data-scarce multiple-attribute cases, merged LoRA experts outperform the baseline expert by up to 5.99 points, demonstrating effective generalization across compounded conditions.

## 1. Introduction

The realization of autonomous driving systems hinges on the ability of their core deep learning models to make stable and appropriate decisions across a wide range of scenarios. This challenge arises in both modular architectures—which progress from perception tasks such as object detection [1] and semantic segmentation [2] to prediction tasks such as pedestrian trajectory prediction [3]—and in end-to-end models [4] that directly map sensor inputs to driving commands. The remarkable performance of modern deep learning models largely depends on the quantity and diversity of their training data. However, current datasets are heavily skewed toward typical scenarios, such as clear and daytime conditions [5,6,7,8]. Data representing long-tail or rare scenarios—such as adverse weather (rainy and snowy) or different times of day (night)—remains scarce. This severe imbalance hampers generalization, leading to significant performance degradation under these rare scenarios. Such degradation is not merely a decline in accuracy on an isolated task; it can cause system-level misjudgments, posing substantial safety risks in real-world deployment. For instance, under conditions where snow accumulation and low illumination overlap, the blurred boundary between the roadway and the sidewalk can cause the system to misidentify the drivable area, leading to the vehicle unintentionally encroaching onto the sidewalk.

This problem is further compounded because scenario attributes such as weather and time-of-day are largely orthogonal. For instance, while data for night or snowy scenarios are already rare, data for multiple-attribute scenarios such as night × snowy are exponentially more difficult to collect. Even comprehensive autonomous driving datasets such as Cityscapes [5], KITTI [6], the Waymo Open Dataset [7], and nuScenes [8] cover only a limited subset of conditions, leaving extreme multiple-attribute scenarios underrepresented. The No Free Lunch theorem [9] suggests that an algorithm’s relative effectiveness depends on the underlying problem distribution; consequently, no single model can be expected to be uniformly superior across all conditions. In autonomous driving, factors such as weather and illumination can substantially shift the observation distribution, making it difficult for a single model to maintain consistently high performance everywhere. Therefore, there is a strong demand for an adaptation method that scalably handles not only single-attribute scenarios but also highly data-scarce multiple-attribute ones.

To address this robustness challenge, a variety of approaches have been explored, including synthetic data generation [10,11,12], image-to-image translation [13,14], unsupervised domain adaptation (UDA) [15,16,17], source-free domain adaptation (SFDA) [18,19,20], and test-time adaptation (TTA) [21,22,23]. However, none of these methods is universally effective; they involve inherent trade-offs among performance, stability, and deployment efficiency.

While single-model adaptation can improve performance under a particular shift, adapting a single model to maintain uniformly strong performance across diverse domains is often challenging, and continued adaptation (e.g., UDA/SFDA-style training or TTA-style online updates) may, depending on the setting, introduce instability or catastrophic forgetting. In contrast, multi-model solutions such as ensembling or mixture-of-experts (MoE) can improve robustness but typically scale compute and memory with the number of experts, which can become a practical constraint for real-time on-vehicle deployment. We therefore seek a lightweight, deployment-friendly multi-expert design that preserves a shared backbone while allowing for efficient switching among specialized experts.

Against this backdrop, we propose a novel adaptation framework based on Low-Rank Adaptation (LoRA) [24], a form of parameter-efficient fine-tuning (PEFT), to overcome these limitations. Our framework keeps the base model’s weights frozen while dynamically attaching lightweight, scenario-specific LoRA experts trained for single-attribute scenarios. This design keeps specialization costs low by storing only small parameter deltas per scenario, enabling efficient expert switching without duplicating the entire model.

However, because training data for compounded (multiple-attribute) conditions is frequently extremely limited, directly fine-tuning a dedicated LoRA expert for each combination can remain unstable and impractical. We therefore leverage LoRA merging to reuse and compose knowledge learned from neighboring single-attribute experts, constructing a merged LoRA for data-scarce multiple-attribute scenarios.

In this work, we focus on semantic segmentation as a critical case study to validate our framework. Our main contributions are summarized as follows:

- A dynamic, scenario-aware inference pipeline: At runtime, we first identify scenario conditions (e.g., weather and time-of-day) from the input image and then dynamically select and apply the optimal pretrained LoRA expert to the frozen base model for robust perception.
- An investigation of LoRA experts for multiple-attribute scenarios: We study the effectiveness of different expert choices under compounded conditions, including single-attribute experts, merged experts, and direct multi-attribute LoRA, and analyze when composing neighboring experts via LoRA merging improves robustness under severe data scarcity.

## 2. Related Works

### 2.1. Single-Model Generalization

A major line of research has focused on enabling a single neural network to operate robustly across diverse scenarios. A common strategy is synthetic data augmentation, where rare or hazardous scenarios are artificially created using simulation datasets and platforms, such as Virtual KITTI [10], CARLA [11], and SHIFT [12], or image-to-image translation models, such as Pix2Pix [14] and CycleGAN [13]. These techniques increase data diversity by generating pseudo-samples for adverse weather or nighttime scenarios that are underrepresented in real-world datasets, thereby improving generalization.

Another prominent direction involves various forms of domain adaptation. Unsupervised domain adaptation (UDA) [15,16,17] traditionally aims to bridge the distribution gap by leveraging both labeled source data and unlabeled target data during training. Source-free domain adaptation (SFDA) [18,19,20] relaxes this requirement, adapting a pretrained source model to the target domain using only the unlabeled target data. Further extending this, test-time adaptation (TTA) [21,22,23] performs rapid, online adjustments during inference, typically adapting the model to incoming test batches without requiring a large target dataset.

While single-model approaches have the advantage of compactness—only one model needs to be stored and deployed—they suffer from inherent limitations. In practice, single-model designs often face a tension between achieving peak performance in specialized domains and maintaining robustness across diverse conditions. This tension is consistent with the No Free Lunch theorems [9], which suggest that effectiveness is distribution-dependent and that a single model is not necessarily consistently advantageous across all operating conditions. Moreover, continual or online adaptation pipelines are vulnerable to catastrophic forgetting [25,26], where previously acquired knowledge is overwritten when adapting to new scenarios, ultimately reducing reliability. These challenges highlight the need for more flexible frameworks that can achieve scalable and stable adaptation across diverse scenarios.

### 2.2. Multi-Model Specialization and Parameter-Efficient Fine-Tuning (PEFT)

To overcome the limitations of single-model generalization, multi-model strategies have been explored, in which multiple expert models are trained independently, each tailored to a specific subset of scenarios (e.g., clear, rainy, or nighttime). These experts can be integrated through mechanisms such as ensemble learning [27,28] or mixture-of-experts (MoE) architectures [29,30]. Ensemble methods combine predictions from independent models to enhance robustness and reduce variance, while MoE architectures dynamically route inputs to the most appropriate expert. Although these strategies perform well on specialized tasks, their practicality in real-world autonomous driving is limited. Increasing the number of experts raises memory and computational requirements that strain on-vehicle real-time resources.

To address these challenges, parameter-efficient fine-tuning (PEFT) has emerged as a promising alternative. PEFT techniques freeze most of the pretrained model’s parameters and update only a small subset, enabling efficient adaptation with minimal computational overhead. Broadly, there are three approaches: (i) adding lightweight components to the existing network to steer internal computations [31,32]; (ii) selectively updating only a restricted subset of the existing parameters, focusing learning on limited elements such as biases or particular layers [33,34]; and (iii) learning compact reparameterizations of weight changes that can be merged back into the base model after training [24,35,36]. Overall, these approaches substantially reduce the number of trainable parameters, mitigate catastrophic forgetting while preserving the general knowledge of the base model, and allow for scenario-specific updates to be easily packaged, swapped, and deployed.

Among various PEFT methods, Low-Rank Adaptation (LoRA) [24] provides an efficient mechanism for scenario adaptation. LoRA assumes that the change in weights during fine-tuning, ΔW, has a low intrinsic rank and approximates it as the product of two smaller matrices, *B* and *A* (ΔW=BA), while keeping the original weights $W_{0}$ frozen. After training, the learned low-rank updates can be algebraically added to the base weights, incurring no additional inference latency. This makes LoRA lightweight, training-efficient, and deployment-friendly compared to adapter-based approaches that alter network architectures. Building on this principle, variants such as AdaLoRA [35], which adaptively allocates low-rank budget, and DoRA [36], which decomposes weights into magnitude and direction, further improve efficiency and stability. Furthermore, the algebraic composability of LoRA-style updates enables the combination of multiple scenario-specific experts at inference time, forming the foundation for scalable model merging discussed in the next subsection.

### 2.3. Merging of Multiple Models

Integrating multiple fine-tuned models to combine their complementary knowledge has become an increasingly active research area. A representative approach is Task Arithmetic [37], which interprets the difference between a pretrained model and its fine-tuned variant as a task vector. By adding or subtracting such task vectors, model capabilities can be edited and combined without additional training. This simple linear operation provides an intuitive and computationally efficient way to merge multiple specialized models.

As another merging method, Spherical Linear Interpolation (SLERP) [38] has been proposed as a geometrically consistent alternative. Instead of simple linear addition, SLERP performs interpolation along the great arc on a hypersphere. Both approaches demonstrate that pretrained models can be combined algebraically to share knowledge and enhance generalization without retraining. Nevertheless, the optimal merging strategy often depends on the specific relationship between tasks or scenarios.

## 3. Methods

**Figure 1:**
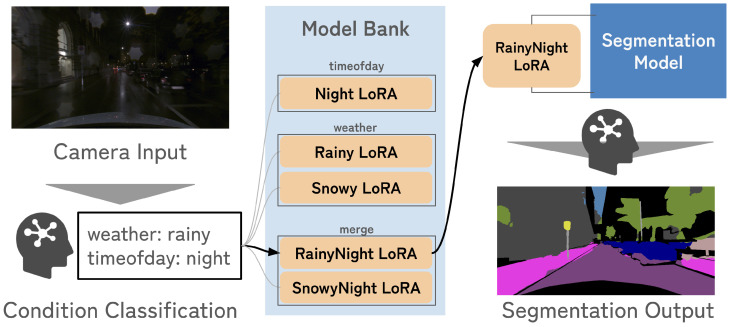
Overview of the proposed scenario-aware inference pipeline. An onboard classifier predicts the current weather and time-of-day, retrieves the corresponding LoRA experts or merged experts, and attaches them to the frozen base model before semantic segmentation.

Our goal is to achieve robust perception across diverse and multiple-attribute scenarios in autonomous driving, where annotated data under such conditions are extremely limited. To this end, we propose a scenario-aware adaptation framework that dynamically integrates multiple lightweight modules depending on the current scenario context. As illustrated in Figure 1, the framework consists of three main components: (1) a condition classification model that identifies scenario attributes such as weather and time-of-day, (2) a set of LoRA-based adaptation experts that provide condition-specific specialization, and (3) a dynamic merging strategy that combines multiple LoRA experts when multiple-attribute scenarios arise. Together, these components enable flexible and efficient adaptation without retraining or increasing inference latency.

### 3.1. Condition Classification

We adopt Swin Transformer V2 [39] as the backbone for this module, building on the shifted-window self-attention introduced in Swin Transformer [40]. We choose Swin Transformer V2 because it achieves high classification accuracy and is strong at capturing global image context. In this architecture, attention is computed within local windows and the window partition is shifted across layers to enable cross-window interaction. Combined with a hierarchical design that merges patches into multi-scale feature maps, the backbone captures fine-grained local cues and broader contextual structure at high resolution with strong efficiency. Since scenario variations in autonomous driving (e.g., changes in weather and time-of-day) are reflected in both local textures and global illumination or context, this architecture serves as a natural and effective choice for our condition classification task.

When an attribute is predicted as undefined, we treat it as absent in the routing logic: if exactly one of weather or time-of-day is undefined, we apply the available single-attribute LoRA; if both are undefined, we fall back to the base model without any LoRA.

### 3.2. LoRA-Based Multi-Model Adaptation

Once the scenario attributes are determined, the corresponding LoRA experts are integrated into the base model to achieve condition-specific adaptation. Low-Rank Adaptation (LoRA) [24] is a parameter-efficient fine-tuning method that assumes the weight update during adaptation has an intrinsically low-rank structure. Given pretrained weights $W_{0}\in R^{d\times k}$, LoRA freezes $W_{0}$ and approximates the fine-tuning update ΔW as follows:

(1) $$\begin{array}{c} \Delta W=BA,\\ A\in R^{r\times k},\;B\in R^{d\times r},\;r\ll \min\left(d,k\right) \end{array}$$

The adapted weights are then expressed as

(2) $$W^{\prime }=W_{0}+\Delta W=W_{0}+BA,$$

where *A* and *B* are learnable low-rank matrices. This formulation substantially reduces the number of trainable parameters while preserving the representational capacity of the base model. Since the LoRA update BA can be directly added to the frozen weights, it introduces no additional inference latency. In our framework, single-attribute (attribute-specific) LoRA experts are independently trained for each attribute value (e.g., night, rainy, and snowy) and stored in a model bank. During inference, the segmentation model is parameterized by the scenario-specific weights $\theta_{0}+\Delta \theta_{s}$, where $\Delta \theta_{s}$ denotes the collection of LoRA updates for scenario *s* (i.e., it aggregates the adapted weights $W^{\prime (s)}=W_{0}+\Delta W^{\left(s\right)}$ across LoRA-instrumented layers).

(3) $$\theta^{\left(s\right)}=\theta_{0}+\Delta \theta_{s}.$$

This design allows for multiple specialized experts to coexist within a unified architecture, enabling scalable adaptation across diverse scenarios.

### 3.3. Dynamic Merging Strategy

When multiple attributes co-occur in the current condition (e.g., night × rainy), the corresponding LoRA experts must be merged to create a unified representation for the multiple-attribute scenario. Previous studies have explored various parameter-merging strategies, including Task Arithmetic [37] and SLERP [38]. Each method exhibits distinct behaviors depending on model characteristics and task relationships, and no single merging strategy is universally optimal. In autonomous driving, where environmental factors interact nonlinearly, the optimal merging method depends on how scenario attributes influence each other.

**Figure 2:**
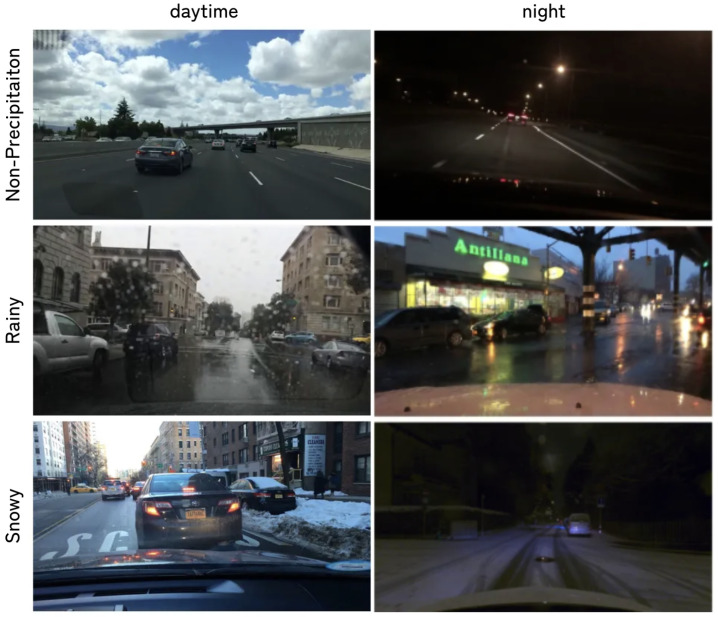
Examples from the constructed dataset (3×2 grid): day and night crossed with non-precipitation, rainy, and snowy conditions.

For instance, as illustrated in Figure 2, night or rainy alone produces limited road reflection, whereas in night × rainy scenes, strong specular highlights emerge due to the interaction between wet road surfaces and artificial light sources. By contrast, in night × snowy, the effects of the individual attributes (night and snowy) are mostly additive, and features emerging only from the combination are relatively scarce.

We analyze two representative merging strategies, Task Arithmetic and SLERP. Intuitively, Task Arithmetic performs a linear combination of update directions, whereas SLERP connects them smoothly along a curved manifold in parameter space.

Task Arithmetic treats each LoRA update as a task vector and merges them through simple linear addition:

(4) $$A_{\mathrm{merge}}=A_{1}+A_{2},\;B_{\mathrm{merge}}=B_{1}+B_{2}$$

(5) $$\Delta W_{\mathrm{merge}}=\lambda \;B_{\mathrm{merge}}A_{\mathrm{merge}}$$

As another method, we also consider SLERP, which interpolates along the great arc on a hypersphere to maintain geometric consistency between parameter directions:

(6) $$\begin{array}{cc} \Delta W_{\mathrm{merge}} & =\frac{\sin((1-\alpha )\theta )}{\sin\theta }\;\Delta W_{1}+\frac{\sin(\alpha \theta )}{\sin\theta }\;\Delta W_{2}, \end{array}$$

where $\theta =\arccos\;\left(\frac{\langle \Delta W_{1},\Delta W_{2}\rangle }{∥\Delta W_{1}∥\;∥\Delta W_{2}∥}\right)$ denotes the angle between the two LoRA updates, and α∈[0,1] is the interpolation coefficient.

## 4. Experiments

### 4.1. Dataset

To demonstrate the effectiveness of our framework across both typical and adverse conditions, we constructed a unified dataset by integrating multiple public benchmarks and then re-partitioning with the scenario attributes required by our method. As sources, we used Cityscapes [5], BDD100K [41], and MUSES [42]. Cityscapes provides high-quality urban annotations primarily collected in Germany (train: 2975 images; val: 500 images), BDD100K offers large-scale diversity across geography, time-of-day, and weather (train: 7000; val: 1000), and MUSES focuses on corner cases such as adverse weather (rainy and snowy) and night, with explicit time-of-day/weather tags (train: 1500; val: 250). Although native image resolutions differ, we resized all images to (height=512,width=1024) for both training and inference to standardize VRAM usage and model inputs. We concatenated the public train/val splits and rebuilt new splits on top of them. Representative examples from our constructed dataset are shown in Figure 2, illustrating the six scenarios formed by day/night crossed with non-precipitation, rainy, and snowy.

#### 4.1.1. Scenario Attribute Assignment and Reconstruction

**Figure 3:**
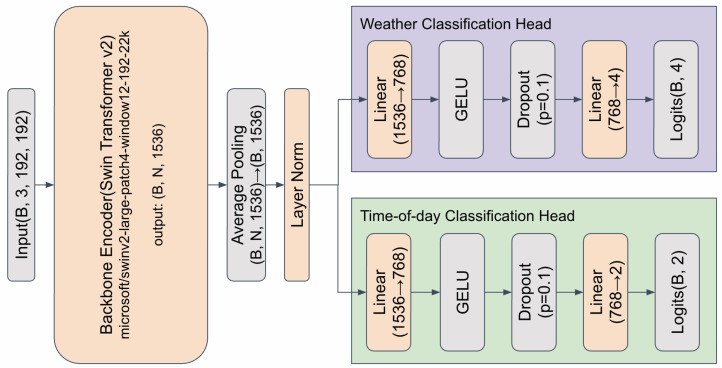
Architecture of the condition-classification model. A Swin Transformer V2 backbone feeds two task-specific heads for weather and time-of-day prediction.

To train attribute-specific (time-of-day and weather) LoRA experts, we assign unified scenario labels to every image using the attribute classifier introduced in Section 3. The classifier is built upon a Swin Transformer V2 [39] backbone pretrained on ImageNet-21k, which is shared across the time-of-day and weather tasks; all parameters of Swin Transformer V2 (including the backbone) are updated during training. Each attribute is predicted independently, and the task-specific head applies LayerNorm to a hidden representation of size *d*, passes it through a fully connected layer that reduces the dimensionality to d/2, follows with GELU and Dropout (0.1), and finally applies a fully connected layer to $C_{t}$ classes to produce logits. The detailed architecture of the condition-classification model is illustrated in Figure 3.

We train the classifier using the existing annotations in MUSES and then run inference with the trained classifier on all images from MUSES, BDD100K, and Cityscapes, attaching time-of-day and weather labels under a consistent definition.

At inference, for each attribute we compute probabilities $p_{t}=\mathrm{softmax}\left(z_{t}\right)$ from the logits and replace the predicted class with undefined whenever $\max_{j}p_{t,j}<\tau$. We fix the threshold as τ=0.5. Decisions are made independently per attribute, so combinations such as timeofday=night and weather=undefined can occur. We introduce undefined to avoid injecting low-confidence pseudo-labels in ambiguous scenarios (e.g., twilight, drizzle vs. fog, or extreme exposure), thereby reducing cross-dataset label noise.

#### 4.1.2. Split Strategy for Evaluation

To rigorously evaluate adaptation to multiple-attribute scenarios via LoRA merging, we adopt a two-step split strategy: (1) we isolate 80% of all night × rainy and night × snowy samples into a dedicated multiple-attribute scenario test split that is completely excluded from training, preventing data leakage and enabling a strict generalization test; (2) we partition the remaining data into train (62.5%), validation (18.75%), and test (18.75%) via stratified sampling that preserves the joint distribution of time-of-day and weather. Consequently, 20% of night × rainy and night × snowy samples remain in the training pool, reflecting realistic development scenarios that include a small fraction of corner cases. This split is intentionally designed to reflect the long-tail nature of compounded conditions: as we consider more scenario attributes (e.g., urban vs. rural, or different countries), the number of possible combinations grows combinatorially, making it increasingly difficult to collect sufficient labeled data for every compound condition. Therefore, we reserve a larger portion of such samples for the test-only split to evaluate generalization under the practically relevant limited-training regime. The resulting dataset split by scenario is summarized in Table 1.

### 4.2. Common Training Setup

All training and evaluation use the unified dataset above with the input resolution fixed to 512×1024. The primary metric is mIoU. We adopt a composite loss that combines cross-entropy with a masked Jaccard term to align optimization with IoU and stabilize training under class imbalance. Unless noted otherwise, we use AdamW and enable automatic mixed precision (AMP). All runs are conducted on a single NVIDIA A100 GPU.

### 4.3. Baseline (Full-Parameter Fine-Tuning; FPFT)

As the baseline, we start from an existing SegFormer-B0 [43] checkpoint that has already been fully fine-tuned on Cityscapes, and we further fine-tune this checkpoint on our unified dataset. We choose SegFormer because it is a transformer-based segmentation architecture that is widely used and exhibits strong accuracy-efficiency trade-offs across benchmarks; its hierarchical MiT encoder and lightweight all-MLP decoder provide competitive performance with practical throughput. We specifically select the B0 variant due to resource constraints (GPU memory and runtime budget) while retaining the architectural characteristics of the SegFormer family. Fine-tuning on the unified dataset is performed for 100 epochs; validation loss decreases consistently and mIoU increases monotonically until saturation toward the end, without signs of overfitting. For subsequent comparisons, we use the epoch-100 checkpoint as the baseline model.

### 4.4. Scenario-Specific LoRA Experts

We freeze the baseline representation and train lightweight LoRA experts specialized for time-of-day and weather. Initialization uses the baseline checkpoint; all non-LoRA parameters remain frozen. The configuration is as follows: rank r=16, α=2r, dropout 0.05, and no bias. LoRA parameters are applied to encoder attention blocks (query, key, value, and dense, proj), the decoder fusion (linear_fuse), and the classifier head (classifier). Each expert is trained on an attribute-filtered subset corresponding to its target condition using AdamW. To improve stability and generalization, we maintain an exponential moving average (EMA) of LoRA parameters and evaluate with the EMA “shadow” weights before restoring the originals. Early stopping is governed by a moving average of the validation loss: the first 100 epochs are a grace period (no stopping); from epoch 101 we track a 50-epoch moving average and stop when it increases for 15 consecutive epochs. The evaluation expert is the EMA-applied checkpoint with the minimum moving-average validation loss.

### 4.5. LoRA Merging Methods

We merge single-attribute LoRA updates per layer while keeping the base weights frozen. Unless otherwise stated, the mixture coefficient is λ=0.5. We evaluate two merging strategies whose mathematical definitions are given in Section 3: (i) Task Arithmetic and (ii) SLERP, which preserves geometric consistency and approximates linear interpolation when the inter-update angle is small. For analysis, we evaluate both methods separately rather than performing automatic selection.

To provide a reference for direct fine-tuning, we additionally train a direct multiple-attribute LoRA (abbreviated as Direct-MA LoRA), i.e., a LoRA model directly fine-tuned on the limited multiple-attribute scenario samples (night × rainy, night × snowy) without merging. This Direct-MA LoRA serves as a baseline to examine whether merging multiple single-attribute experts can outperform direct training on scarce multiple-attribute data.

## 5. Results

### 5.1. Condition Classifier Performance and Its Impact on Re-Partitioning

The re-partitioning of the unified dataset relies on the weather and time-of-day labels estimated by the condition classifier. Therefore, if the classifier accuracy is low, systematic errors can enter the construction of the training data for single-condition LoRA and the evaluation data for multi-condition scenarios, which can bias subsequent comparisons. In this section, we verify the accuracy of weather and time-of-day classification on MUSES, which provides ground-truth labels.

Table 2 reports the accuracy of the condition classifier on the MUSES test split. The weather classifier achieves 98.86% accuracy and the time-of-day classifier achieves 100.00%, indicating very high performance. Because Cityscapes and BDD100K do not provide ground-truth condition labels, we cannot directly measure classification accuracy on those sources; however, at least on MUSES, misclassifications are extremely limited. Therefore, the impact of condition classification errors on the re-partitioning of the unified dataset used in this study is expected to be relatively small.

### 5.2. Single-Attribute Scenario Adaptation

We compare the FPFT baseline with single-attribute LoRA experts specialized for each scenario (snowy, rainy, and night). Each expert is trained with five independent runs using different random seeds, and we report the mean and standard deviation of the mIoU (Table 3).

LoRA-based adaptation consistently outperforms the FPFT baseline across all single-attribute scenarios. The small standard deviations indicate that these improvements are stable across random initializations rather than seed-specific fluctuations.

### 5.3. Parameter Count Comparison

Table 4 reports the parameter counts of the baseline SegFormer-B0 model and the single-condition LoRA. Compared with the 3,719,540-parameter baseline, single-condition LoRA has 227,936 parameters, which is about 6.1% of the baseline. Therefore, sharing the base model and storing only LoRA per condition is advantageous for scalable adaptation across multiple conditions.

These results confirm that training a LoRA per single condition can stably improve condition-specific performance while keeping parameter growth far smaller than that of the base model.

### 5.4. Multiple-Attribute Scenario Adaptation via LoRA Merging

We evaluate generalization to unseen scenario combinations (multiple-attribute conditions: night × snowy and night × rainy) using the two merging strategies introduced in Section 4.5. Single-attribute LoRAs are trained with five seeds and merged in a seed-paired fashion; we report the mean mIoU and standard deviation. As a reference, we also include direct multiple-attribute LoRA (abbreviated as Direct-MA LoRA), which is directly fine-tuned on limited multiple-attribute samples (night × snowy and night × rainy) without merging.

We evaluate LoRA merging for multiple-attribute scenarios (night × snowy and night × rainy). On night × snowy, the Task Arithmetic merge improves performance over the baseline by +4.19 mIoU points (Table 5), while on night × rainy, SLERP achieves a larger gain of +5.99 mIoU points over the baseline (Table 6). These results suggest that composing single-attribute LoRA experts can effectively leverage complementary knowledge and lead to substantial performance improvements under data-scarce multiple-attribute conditions. In contrast, Direct-MA LoRA, which is directly fine-tuned on limited multiple-attribute samples, yields only limited improvements over the baseline in both settings, indicating that direct adaptation alone may be insufficient when training data for compounded conditions is scarce.

We further conducted a comprehensive evaluation of the proposed method as an integrated pipeline. This assessment covers the entire dataset, encompassing all scenarios within the standard test split as well as the multiple-attribute test-only split. We compared five distinct inference strategies to verify the effectiveness of adaptive model selection. The first two strategies involve the uniform application of either the FPFT baseline or the All LoRA model across all conditions. The third strategy is a conditional selection approach that applies the corresponding single-attribute LoRA for single adverse conditions; for multiple-attribute scenarios, it selects the single expert that demonstrated higher individual accuracy. The fourth strategy, representing our optimized pipeline, similarly employs single-attribute LoRA for single conditions but adopts the optimal inference method for combined conditions, specifically using SLERP for night × rainy and the night expert for night × snowy. The fifth strategy incorporates the specialized Direct-MA LoRA; it utilizes the All LoRA model for normal conditions, single-attribute LoRA for single adverse conditions, and the Direct-MA LoRA for multiple-attribute scenarios. The overall evaluation on the entire test set, including multiple-attribute scenarios, is summarized in Table 7.

Despite consistent gains over FPFT, the absolute margins across LoRA-based strategies remain relatively modest. This is partly attributable to the inherent difficulty of interpreting low-illumination scenes from standard RGB camera imagery alone, where ambiguity and sensor noise can limit separability among methods. While our results clearly indicate that LoRA-based adaptation is broadly effective, identifying the optimal merging/selection strategy in a condition-dependent manner likely requires further study with richer datasets and/or additional sensing cues.

### 5.5. Qualitative Evaluation

**Figure 4:**
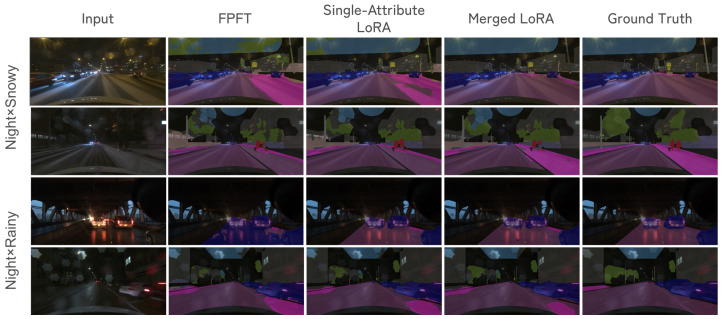
Qualitative results on night × snowy (**top**) and night × rainy (**bottom**). (**left** to **right**) Input, FPFT, single-attribute LoRA (best of weather/time-of-day), merged LoRA (best of Task Arithmetic/SLERP), and ground truth.

Qualitative results illustrate concrete improvements achieved by the merged LoRA models (Figure 4). In night × snowy, both the baseline and the single-attribute LoRA mislabel road regions as sidewalk, whereas the merged LoRA correctly restores the drivable area. In night × rainy, specular reflections on wet asphalt lead to false-positive vehicle detections in the baseline output; the merged LoRA suppresses these spurious vehicle regions.

## 6. Conclusions

In this work, we tackled robust perception for autonomous driving under multiple-attribute scenarios where adverse factors co-occur under scarce data. We proposed an adaptive framework that dynamically merges scenario-specific LoRA modules, formulating multiple-attribute scenario adaptation as composition in parameter space. Depending on the environmental attributes, the system switches between single-attribute LoRA and merged LoRA, applying the appropriate parameters to the base model. On a unified corpus from MUSES, BDD100K, and Cityscapes, our approach improves the mIoU over FPFT and single-scenario experts and enables generalization to unseen combinations. Qualitative results show reduced artifacts and more stable predictions under adverse conditions. This composability preserves the frozen base model and incurs no inference-time overhead in practical systems. Looking ahead, we will move beyond discrete labels toward continuous latent representations that capture fine-grained variations. Leveraging these attributes to guide merging and conflict estimation, we aim to establish a label-independent framework for robust adaptation across perception and, ultimately, decision-making.

## Figures and Tables

**Table 1:** Dataset split by scenario. Non-precipitation aggregates Clear, Foggy, and Undefined. The multiple-attribute scenarios (test-only split) include night × rainy and night × snowy.

Scenario	Train	Val	Test	Multiple-Attribute Scenarios (Test-Only Split)
day × non-precipitation	6975	2093	2093	–
day × rainy	164	49	49	–
day × snowy	446	134	134	–
night × non-precipitation	443	133	134	–
night × rainy	23	7	6	144
night × snowy	25	7	8	158
Total	8076	2423	2424	302

**Table 2:** Condition classifier performance measured on the MUSES test split.

Classification Task	Accuracy (%)
Weather classification	98.86
Time-of-day classification	100.00

**Table 3:** Comparison between FPFT and single-attribute LoRA experts (mean over five seeds).

Scenario	FPFT	LoRA (Mean ± Std)	**Δ** mIoU (pt)
Snowy	59.47	60.60 ± 0.27	+1.13
Rainy	53.80	57.03 ± 0.26	+3.23
Night	46.77	48.56 ± 0.52	+1.79

**Table 4:** Parameter count comparison between the Baseline and Single-Condition LoRA.

Model	Parameter Count	Relative to Baseline
Baseline (SegFormer-B0)	3,719,540	–
Single-Condition LoRA	227,936	0.061

**Table 5:** The night × snowy split relative to the baseline. Best values are underlined.

Method	mIoU (Mean ± Std)	**Δ** vs. Baseline (pt)
FPFT	41.50	–
All LoRA	44.64 ± 0.38	+3.14
Snowy LoRA	42.99 ± 0.35	+1.49
Night LoRA	45.95 ± 0.75	+4.45
Task Arithmetic	45.69 ± 1.12	+4.19
SLERP	45.44 ± 0.36	+3.94
Direct-MA LoRA	42.10 ± 0.39	+0.60

**Table 6:** The night × rainy split relative to the baseline. Best values are underlined.

Method	mIoU (Mean ± Std)	**Δ** vs. Baseline (pt)
FPFT	37.95	–
All LoRA	42.21 ± 0.11	+4.26
Rainy LoRA	43.07 ± 0.20	+5.12
Night LoRA	42.62 ± 0.39	+4.67
Task Arithmetic	40.90 ± 0.92	+2.95
SLERP	43.94 ± 0.33	+5.99
Direct-MA LoRA	39.37 ± 0.88	+1.42

**Table 7:** Overall evaluation on the entire test set including multiple-attribute scenarios. Best values are underlined.

Method	mIoU (Mean ± Std)	**Δ** vs. FPFT (pt)
FPFT	61.76	–
All LoRA	63.03 ± 0.05	+1.27
Single-Attribute LoRA	63.25 ± 0.06	+1.49
Our Pipeline	63.32 ± 0.06	+1.54
Direct-MA LoRA	62.72 ± 0.07	+0.96

## Data Availability

The data used in this study are obtained from publicly available datasets: Cityscapes (https://www.cityscapes-dataset.com/), BDD100K (http://bdd-data.berkeley.edu/), and MUSES (https://muses.vision.ee.ethz.ch/).
